# Neighborhood Alcohol Outlet Density, Historical Redlining, and Violent Crime in NYC 2014–2018

**DOI:** 10.3390/ijerph20043212

**Published:** 2023-02-12

**Authors:** Sean J. Haley, Shari J. Jardine, Elizabeth A. Kelvin, Christopher Herrmann, Andrew R. Maroko

**Affiliations:** 1 Department of Health Policy and Management, CUNY Graduate School of Public Health and Health Policy, City University of New York, New York, NY 10027, USA; 2 Department of Community Health and Social Sciences, CUNY Graduate School of Public Health and Health Policy, City University of New York, New York, NY 10027, USA; 3 Department of Epidemiology and Biostatistics, CUNY Graduate School of Public Health and Health Policy, City University of New York, New York, NY 10019, USA; 4 CUNY Institute for Implementation Science in Population Health, City University of New York, New York, NY 10027, USA; 5 Department of Occupational Health, Epidemiology & Prevention, Donald and Barbara Zucker School of Medicine at Hofstra University/Northwell Health, Hempstead, NY 11549, USA; 6 Department of Law & Police Science, John Jay College, City University of New York, New York, NY 10027, USA; 7 Department of Environmental, Occupational, and Geospatial Health Sciences, CUNY Graduate School of Public Health and Health Policy, City University of New York, New York, NY 10027, USA

**Keywords:** alcohol, policy, density, redlining, violent crime, policy

## Abstract

Alcohol outlets tend to cluster in lower income neighborhoods and do so disproportionately in areas with more residents of color. This study explores the association between on- and off-premise alcohol outlet density and history of redlining with violent crime in New York City between 2014 and 2018. Alcohol outlet density was calculated using a spatial accessibility index. Multivariable linear regression models assess associations between the history of redlining, on-premise and off-premise alcohol outlet density with serious crime. Each unit increase in on- and off-premise alcohol density was associated with a significant increase in violent crime (β = 3.1, *p* < 0.001 on-premise and β = 33.5, *p* < 0.001 off premise). In stratified models (redlined vs not redlined community block groups) the association between off-premise alcohol outlet density and violent crime density was stronger in communities with a history of redlining compared to those without redlining (β = 42.4, *p* < 0.001 versus β = 30.9, *p* < 0.001, respectively). However, on-premise alcohol outlet density was only significantly associated with violent crime in communities without a history of redlining (β = 3.6, *p* < 0.001). The violent crime experienced by formerly redlined communities in New York City is likely related to a legacy of racialized housing policies and may be associated with state policies that allow for high neighborhood alcohol outlet density.

## 1. Introduction

In the two decades prior to the onset of the COVID-19 pandemic, there was a significant net increase in U.S. alcohol consumption of approximately 3% per decade. Binge drinking increased during the same period by 7.5% per decade [1]. Between 2006 and 2014, there was a 62% increase in alcohol related emergency room visits [2]. Furthermore, according to the National Institute on Alcohol Abuse and Alcoholism, there was a doubling of alcohol-related deaths for those aged 16 and above from 1999 to 2017 (from 35,914 to 72,558), such that the overall age-adjusted death rate increased by 50.9% (from 16.9 to 25.5 per 100,000). Rates increased for all age groups, except for those 75 and older, and increased for all racial and ethnic groups, except among Hispanic males and non-Hispanic Blacks whose rates dipped initially then increased [3].

For over a decade, the Community Preventive Services Task Force has recommended reducing alcohol outlet density to decrease alcohol-related harms [4]. Societal harms associated with alcohol outlet density include greater youth access and underage drinking [5,6], as well as increased instances of violent crime [7]. In New York City (NYC), alcohol outlet density was associated with increased prevalence of alcohol use disorders [8], and cyclist and pedestrian death by a motor vehicle [9]. Off-premise alcohol retail store and on-premise restaurant alcohol outlet density in NYC were significantly associated with street robbery and aggravated assault while on-premise bars/taverns were not [10]. Regulating alcohol outlet density through the reduction or limitation of alcohol licenses remains an important strategy to reduce alcohol-related harms [4].

Consistent with structural frameworks of disease causation [11], alcohol outlets tend to cluster in lower income neighborhoods and do so disproportionately in high poverty areas with more Black, Indigenous, and other residents of color [12,13]. In addition, people living with lower socio-economic status experience nearly twice the mortality from alcohol-attributable causes compared to all other causes of mortality [14,15]. Previous studies found associations between alcohol outlet density and poverty [7,16], as well as associations between alcohol outlet density and the legacy of discriminatory housing practices, including ‘redlining’ [17].

As a part of the New Deal initiatives during the Great Depression, the Federal Housing Authority supervised the sale of homes constructed with federal dollars and enacted policies that prohibited their sale to ‘inharmonious racial groups’, citing a potential loss of property values, which would place these federally insured loans at risk of default [18,19]. The Home Owners’ Loan Corporation provided risk assessments to the Federal Housing Authority, including color-coded maps. ‘Undesirable’ neighborhoods were given the letter grade ‘D’ and colored in red [19,20]. Consequently, federally insured loans included ‘restrictive covenant’ clauses within mortgage contracts and deeds that prohibited sale or resale to Black families [19], effectively barring Black families from gaining or passing wealth achieved from home ownership to future generations [21]. As such, the Home Owners’ Loan Corporation maps serve as a proxy for past racialized government policies. Although outlawed in the 1960s, the laws serve as codified discriminatory policies and are separate from the indicators of the policies’ consequences, including racial concentration end economic sedimentation [22]. This study aims to: (1) explore the association of on- and off-premise alcohol outlet density and the history of redlining with violent crime in New York City between 2014 and 2018, and (2) assess whether the associations between alcohol outlet density and violent crime are modified by a history of redlining.

## 2. Materials and Methods

### 2.1. Alcohol Outlet Density at the Census Block Group (CBG) Level

Point location data for alcohol outlets were extracted from the New York State Liquor Authority database by utilizing the public license query feature [23]. To minimize the effect of licenses that were newly approved just prior to the start or the end of the study period (2014–2018), licenses with approval dates less than six months prior to the start of 2014, and approval dates within the last six months of 2018 were excluded. License location data were matched to address points using the ArcGIS World Geocoder (n = 11,774). This included both on-premise (n = 6606) and off-premise (n = 5168) alcohol outlets. Wholesale (n = 16), manufacturing (n = 11), and seasonal (n = 27) licenses were excluded. Alcohol outlets with incomplete, unmatched, and/or duplicate addresses were excluded (n = 65, 0.6%).

To calculate alcohol outlet density, the CDC recommends several methods, which fall into four broad categories: count-based, container-based, distance-based, and spatial access-based [24]. This study utilized a spatial accessibility index, which is better suited to assess clustering, measure exposed populations, address access potential, and evaluate harms when compared to simpler container- and distance-based approaches [25]. The spatial accessibility index was calculated by first specifying a “choice set” (the number of outlets used to assess population exposure). The CDC recommends values between five and nine [24]. In this study, we selected nine due to the relatively high population density and concentration of outlets in most NYC boroughs. The Euclidean distance from each CBG centroid (representing population centers) and the nearest nine outlets was then determined. The spatial access score/alcohol outlet density was then calculated as the sum of the inverse distances for all nine alcohol outlets per CBG. As such, the shorter distances to the nearest outlets in a CBG result in larger alcohol outlet density scores.

### 2.2. Violent Crime

Violent crime data were obtained from the New York City Police Department for the years 2014–2018. Violent crime was defined as murder, shooting victim, rape, robbery, or aggravated assault, as these categories are more consistently reported to law enforcement agencies. In New York City, violent crime points were geocoded to the nearest intersection or the midsection of street segments [24]. The geo-coded points were then aggregated to CBG and divided by area to calculate violent crime density (crimes per mi^2^) to match the other datasets for analysis.

### 2.3. Redlining

Redlining data were obtained from the Mapping Inequality database and capture data produced between 1935 and 1940 [26]. Using Arc GIS, previously redlined areas were overlayed with current maps of the five boroughs of New York City to align them to current CBG boundaries. CBGs in which greater than 50% of the area was previously redlined were categorized as having a history of redlining.

### 2.4. Neighborhood Demographics

CBG population characteristics were obtained from the 2018 American Community Survey (ACS) 5-year estimates for years 2014–2018 [27]. The population is described in terms of the percent with income below the poverty federal poverty line, percent of adults ≥25 years old who did not graduate from high school, population density per mile squared, percent of the population identifying as non-Hispanic Black, percent of the population identifying as Hispanic/Latino, percent of the population that moved in since 2015, percent of housing units that were vacant, and the percent of housing units which were owner-occupied. New York City CBGs served as the unit of analysis. Ninety-six CBGs (populations of fewer than 100 residents including airports, commercial areas, and parks), were excluded from the analysis.

### 2.5. Statistical Analysis

We described the communities overall and stratified on whether they had a history of redlining. The statistical significance of differences in community characteristics by redlining history was assessed with a Wilcoxon rank sum test. We mapped the distribution of the variables of primary interest (density of on-premise, off-premise alcohol outlets, and crime), indicating which neighborhoods had a history of redlining in New York City using ArcGIS Pro version 2.9 [28]. We then ran crude and multivariable linear regression models to assess the crude and adjusted associations between history of redlining, on-premise alcohol outlet density, and off-premise alcohol outlet density with density of serious crime. The multivariable model included all the characteristics of the communities described above. We then added interaction terms for redlining history*on-premise alcohol outlet density and redlining history*off-premise alcohol outlet density to the multivariable model with the understanding that if either interaction term was significant, we would run the multivariable model stratified on redlining history to assess effect modification (i.e., how the association between on- and off-premise alcohol outlet density differs between communities with a history of redlining versus those without such a legacy). All analyses were conducted in R and significance set at α = 0.05 for main effects and α = 0.1 for effect modification, due to the lower statistical power associated with testing interaction.

## 3. Results

### 3.1. Description of the Communities

**Figure 1:**
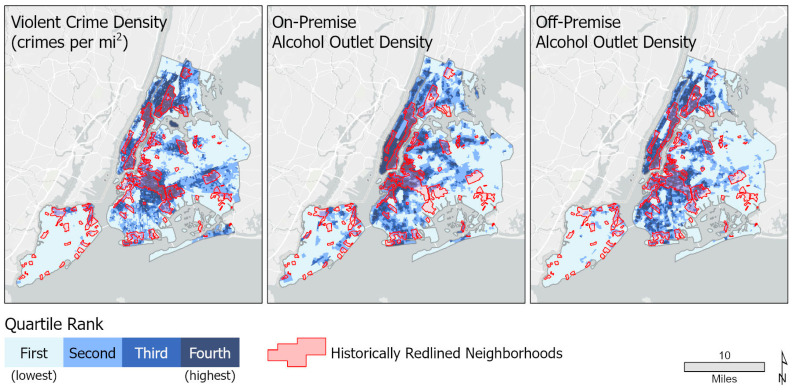
Distribution of violent crime density, on-, and off-premise alcohol outlet density/access score in redlined and non-redlined neighborhoods in New York City.

A total of 6198 CBGs were included in the analyses. Overall, 28.2% of NYC CBGs had at least 50% of their area in historically redlined neighborhoods. The average number of violent crimes per square mile was 2012 in historically redlined CBGs compared to 1166.5 in non-redlined communities (*p* < 0.001). The mean alcohol outlet density for both on- and off-premise outlets was significantly higher in redlined CBGs (14.0 versus 8.7, *p* < 0.001 and 13.0 versus 9.6, *p* < 0.001, respectively) (Figure 1).

Other CBG variables, including population density, percent below the federal poverty line, percent of adults 25 years or older without a high school degree, percent of the population identifying as Hispanic/Latino or non-Hispanic Black, percent of the population that moved in since 2015, and percent of homes that were vacant, were all significantly greater in redlined communities. The percent of owner-occupied homes was significantly lower in redlined communities (24.7% versus 39.7%, *p* < 0.001) (Table 1).

### 3.2. Linear Regression Results

In the crude models, CBGs with a history of redlining had on average 845.8 more violent crimes per square mile than neighborhoods without a history of redlining (*p* < 0.001). Each unit increase in the number of on-premise alcohol outlets per square mile was associated with an increase of 6.9 violent crimes per square mile (*p* < 0.001), and each unit increase in the number of off-premise alcohol outlets per square mile was associated with an increase of 88.6 crimes per square mile (*p* < 0.001) (Table 2). 

After adjusting for community characteristics in the multivariate model, the associations were attenuated but remained significant. Communities with a history of redlining experienced 205.8 more crimes per square mile, on average, than communities without a history of redlining (*p* < 0.001). Each unit increase in on- and off-premise alcohol density was associated with a significant increase in violent crime, although association was stronger for off-premise density (β = 3.1, *p* < 0.001 and β = 33.5, *p* < 0.001, respectively) (Table 2). In a test for collinearity, the variable inflation factor (VIF) for the redlining variable was just over 1.1 and VIFs for all the other variables had values below 2.2, well below recommended thresholds.

### 3.3. Effect Modification of the Association between On- and Off-Premise Alcohol Outlet Density and Violent Crime Density by History of Redlining

When interaction terms for history of redlining*alcohol outlet density were added to the multivariate model, both redlining*off-premise alcohol outlet density and redlining*on-premise outlet density were statistically significant at our a priori α = 0.1 (interaction term *p* < 0.001 and *p* = 0.090, respectively). We ran the multivariable model stratified on the history of redlining and found that the strength of the association between alcohol outlet density and violent crime density varied by history of redlining, but the direction of the variation differed for on-versus off-premise outlets. As hypothesized, the association between off-premise alcohol outlet density and violent crime density was stronger in communities with a history of redlining compared to those without (β = 42.4, *p* < 0.001 versus β = 30.9, *p* < 0.001, respectively). However, on-premise alcohol outlet density was not associated with violent crime in formerly redlined neighborhoods. Rather, the association between on-premise alcohol outlet density and violent crime density was only significant in communities without a history of redlining compared to those with such a legacy (β = 3.6, *p* < 0.001. versus β = 2.8, *p* = 0.170, respectively) (Table 3).

## 4. Discussion

We found that the distribution of on- and off-premise alcohol outlets and of violent crime was denser in communities with a history of redlining. Furthermore, there was a positive association between density of both on- and off-premise alcohol outlets and violent crime. Importantly, the structural effects of redlining are maintained when current socio-economic indicators are adjusted for, suggesting that formerly redlined areas continue to be associated with crime independent of the current SES indicators that were added to the model. In addition, in the stratified model separating historically redlined neighborhoods from other neighborhoods, we found that the association between off-premise (but not on-premise) alcohol outlet density and violent crime density was significantly stronger in communities with a history of redlining compared to those without this history.

Previous studies found similar associations between alcohol outlet density and violent crime [7,10,14], and alcohol density and redlining [15]. Gorman et al. (2001) assessed the association between alcohol outlet density (measured as total outlets per 100 population) and violent crime in Camden, New Jersey, controlling for poverty and other population characteristics [16]. The study found that total (on-premise + off-premise) alcohol outlet density contributed significantly to violent crime within block groups [16]. Trangenstein et al. (2018) explored the association between access to alcohol outlets and violent crime in Baltimore MD, with attention to outlet characteristics and types of crime [7]. Using a spatial accessibility index, the Trangenstein study found a positive relationship between alcohol outlet density and violent crime. Specifically, the authors found that each 10% increase in alcohol outlet access was associated with a 4.2% increase in violent crime exposure. The authors also identified differential effects, such that a 10% increase in access to off-premise outlets and combined off- and on-premise outlets had a greater association with violent crime than on-premise outlets [7]. In a subsequent Baltimore study, Trangenstein et al. (2020) examined the association between CBG characteristics and alcohol outlet clusters by type of alcohol outlet. The authors found that CBGs that were redlined had 7.3 times the odds of being in an off-premise cluster, 8.1 times the odds of being in an on-premise cluster, and 8.6 times the odds of being in a combined (on- and off-premise) cluster [17].

Feng et al. also used spatial adjustment to assess the association between alcohol outlets and street robberies and aggravated assaults in NYC. They found that among nine categories of alcohol outlets, two on-premise (eateries and restaurants) and two off-premise alcohol outlets (grocery stores and alcohol retail stores) were associated with aggravated assault. In addition, three on-premise (e.g., eateries, bars/taverns, and restaurants) and three off-premise alcohol outlets (e.g., grocery stores, alcohol retail stores, and drug stores) were associated with street robberies. While grocery stores were associated with robberies and assault in all five boroughs, three on-premise venues (e.g., night clubs, hotels, and other eateries) were not associated with robberies or assaults in any borough [10].

Interestingly, while we found that the association between density of off-premise alcohol outlets and violent crime was modified by redlining, the direction of the effect modification for on-premise alcohol outlets was such that the association between on-premise alcohol outlet density and violent crime density was only modified in communities without historical redlining. Our finding may be related to neighborhood variations in economic composition, including rapid neighborhood gentrification in NYC, or to variation in the types of on-premise outlets (hotels vs. nightclubs) within those neighborhoods [15]. Still, although outlawed in the 1960s, redlining codified discriminatory housing policies and continues to be associated with a myriad of health conditions in NYC and elsewhere, suggesting that it has long-lasting impacts [22].

In NY State, Chapter 478 of the Laws of 1934 created the State Liquor Authority and the Division of Alcoholic Beverage Control. According to the law, the State Liquor Authority was established to “regulate and control the manufacture and distribution within the state of alcoholic beverages for the purpose of fostering and promoting temperance in their consumption and respect for and obedience to law; for the primary purpose of promoting health, welfare and safety of the people of the state, and, to the extent possible, supporting economic growth…” The statute also authorizes the State Liquor Authority to “determine whether public convenience and advantage will be promoted by the issuance of licenses to traffic in alcoholic beverages… and to carry out the increase or decrease in the number thereof and the location of premises licensed… in the public interest” [29]. In keeping with its health mandate, results from this paper suggest that the State Liquor Authority might consider limiting the number or licenses in neighborhoods with high outlet density.

Alcohol license applicants in NY State must notify their respective municipalities 30 days in advance of submitting the application. In NYC, the city notifies the appropriate community board. Even though communities can submit a recommendation opposing the alcohol license application, the recommendations are not binding. In 2022 it was revealed that the State Liquor Authority receives 75,000 applications every year and that the average review time is 26 weeks [30,31]. Rather than calling for a review of alcohol outlet density in the face of increasing alcohol related harms, in 2022, Governor Hochul proposed increasing the agency’s budget by 2 million dollars to expedite alcohol application processing [31]. Given the results of this and other studies that suggest strong associations between alcohol outlet density and violent crime in NY [7,10,14], the governor’s budget request does not appear to align with the NY State Liquor Authority’s stated mission of promoting health and safety.

This study has limitations. The analyses used data aggregated to the CBG, and thus the results can only be interpreted as applying to the CBG and not to the individuals living within these communities (i.e., the ecological fallacy whereby population-level correlations are assumed to parallel individual-level correlations) [32]. Although we used a spatial accessibility index as recommended [23], and our analysis relied on Euclidean distances, it is possible that the use of network distances potentially produced slightly different results. As other researchers suggested, there is tremendous variation within categories of on- and off-premise alcohol outlets related to outlet size, capacity, how alcohol is consumed, and whether alcohol consumption by those in the neighborhood is directly related to the violence committed in the defined geographic areas [33]. In addition, the analyses relied on the most recent violent crime data available to the researchers at the time of the study, and the analyses were cross-sectional, such that we cannot determine causation. Furthermore, it is possible that we failed to adjust for all confounders, for example, we did not control for sociodemographic variables in the year 1940 that reflected neighborhood composition to control for differences that pre-dated redlining maps [34]. We might also point out that this analysis was conducted with data from New York City before the COVID-19 pandemic, and it is unclear whether similar associations would be found following the pandemic or in other locations.

Findings from this study add to the growing literature related to the persistent negative health consequences of structural racial discrimination. Although there are a myriad of potential pathways, the effects of legally codified discrimination appear to have sedimentary health effects on the populations who remain [22]. Our findings suggest that the persistent health effects of redlining will not be easily reversed, but that reducing the density of alcohol outlets may serve to ameliorate at least one set of health consequences.

## 5. Conclusions

Our results suggest that high concentrations of alcohol outlets are associated with violent crimes within low-income neighborhoods. In addition, racialized housing practices appear to have a persistent negative impact on neighborhoods long after such practices are formally abolished. Reducing the concentration of alcohol outlets may be one strategy to reduce violent crime in NYC neighborhoods, the effects of which may be stronger within formerly redlined communities. As such, initiatives addressing neighborhood planning, zoning, and licensing remain the effective approaches to reduce socioeconomic inequalities for alcohol-attributable outcomes [35].

## Figures and Tables

**Table 1:** Description of the Communities overall and by history of redlining.

Variable	Overall	Redline Neighborhood	Non-Redlined Neighborhood	Wilcoxon Rank Sum *p*-Value
Census block groups, n (%)	6198 (100.00)	1750 (28.23)	4448 (71.77)	NA
Density of violent crime per mile^2^				<0.001
Mean (SD)	1405.28 (1759.80)	2012.25 (1912.20)	1166.47 (1635.68)	
Median (min, max)	728.85 [0.00, 19720.90]	1462.80 [0.00, 16527.10]	542.85 [0.00, 19720.90]	
On-premises alcohol outlet density				<0.001
Mean (SD)	10.17 (17.11)	14.00 (20.47)	8.67 (15.33)	
Median (min, max)	6.39 [0.78, 786.60]	9.07 [0.97, 454.70]	5.80 [0.78, 786.60]	
Off-premise alcohol outlet density				
Mean (SD)	10.55 (7.20)	12.98 (7.02)	9.60 (7.05)	<0.001
Median (min, max)	9.41 [0.95, 217.55]	12.23 [1.30, 119.34]	8.21 [0.95, 217.55]	
Percent of the population living below the federal poverty line				<0.001
Mean (SD)	18.26 (15.09)	22.42 (17.14)	16.62 (13.87)	
Median (min, max)	14.10 [0.00, 93.50]	18.80 [0.00, 93.50]	12.90 [0.00, 91.60]	
Percent of the population without a high school degree				0.003
Mean (SD)	18.48 (13.80)	19.87 (15.52)	17.93 (13.03)	
Median (min, max)	16.00 [0.00, 80.60]	17.55 [0.00, 80.60]	15.40 [0.00, 76.30]	
Population density per mile^2^				<0.001
Mean (SD)	65596.56 (52965.28)	79200.29 (54825.55)	60244.37 (51240.69)	
Median (min, max)	50829.50 [79.40, 518070.40]	65695.85 [659.10, 490690.70]	45193.00 [79.40, 518070.40]	
Percent of the population identifying as non-Hispanic Black				<0.001
Mean (SD)	21.89 (28.35)	27.61 (29.09)	19.64 (27.73)	
Median (min, max)	6.50 [0.00, 100.00]	15.70 [0.00, 100.00]	5.00 [0.00, 100.00]	
Percent of the population identifying as Hispanic/Latino				0.005
Mean (SD)	27.88 (25.01)	29.35 (25.50)	27.31 (24.79)	
Median (min, max)	19.10 [0.00, 100.00]	21.05 [0.00, 98.30]	18.30 [0.00, 100.00]	
Percent of the population that moved in since 2015				<0.001
Mean (SD)	13.20 (9.22)	14.93 (9.90)	12.52 (8.85)	
Median (min, max)	11.97 [0.00, 81.25]	13.84 [0.00, 59.22]	11.32 [0.00, 81.25]	
Percent of the housing that is vacant				<0.001
Mean (SD)	8.61 (8.13)	9.51 (8.37)	8.26 (8.01)	
Median (min, max)	7.00 [0.00, 67.10]	7.70 [0.00, 51.90]	6.80 [0.00, 67.10]	
Percent of homes owned by the residents				<0.001
Mean (SD)	35.43 (28.07)	24.66 (22.82)	39.67 (28.81)	
Median (min, max)	30.81 [0.00, 100.00]	19.05 [0.00, 100.00]	36.97 [0.00, 100.00]	

**Table 2:** Linear regression models examining the association of redlining, on-premise and off-premise alcohol outlet density, and covariates with crime.

	Crude Models (n = 6198 for All Models)			Multivariable Model (n = 6198)			
Variable	Beta	95% Confidence Interval	*p*-Value	Beta	95% Confidence Interval	*p*-Value	*p*-Value for Interaction W/Redlining Added to Multivariable Model
History of redlining	845.77	750.73, 940.82	<0.001	205.77	128.68, 282.85	<0.001	
On-premise alcohol outlet density	6.92	4.36, 9.47	<0.001	3.08	0.97, 5.18	<0.001	0.090
Off-premise alcohol outlet density	88.62	82.95, 94.29	<0.001	33.50	28.08, 38.92	<0.001	<0.001
Percent of the population below the federal poverty line				15.82	12.83, 18.8	<0.001	
Percent of the population without a high school degree				7.96	4.57, 11.35	<0.001	
Population density per mile^2^				0.01	0.01, 0.01	<0.001	
Percent of the population identifying as non-Hispanic Black				15.31	14.08, 16.55	<0.001	
Percent of the population identifying as Hispanic/Latino				16.61	14.85, 18.38	<0.001	
Percent of the population that moved in since 2015				−2.40	−6.36, 1.55	0.230	
Percent of the housing that is vacant				6.34	2.14, 10.55	<0.001	
Percent of homes owned by the resident				−7.45	−9.19, −5.71	<0.001	

**Table 3:** Multivariable linear regression model stratified on history of redlining.

	Redlined Neighborhoods (n = 1750)			Non-Redlined Neighborhoods (n = 4448)		
Variable	Beta	95% Confidence Interval	*p*-Value	Beta	95% Confidence Interval	*p*-Value
On-premises alcohol outlet density	2.81	−1.17, 6.79	0.170	3.63	1.14, 6.11	<0.001
Off-premise alcohol outlet density	42.36	30.87, 53.85	<0.001	30.94	24.94, 36.94	<0.001
Percent of the population living below the federal poverty line	15.21	9.36, 21.06	<0.001	14.31	10.86, 17.76	<0.001
Percent of the population without a high school degree	4.98	−2.07, 12.02	0.170	8.49	4.71, 12.27	<0.001
Population density per mile^2^	0.01	0.01, 0.01	<0.001	0.01	0.01, 0.01	<0.001
Percent of the population identifying as non-Hispanic Black	20.85	18.03, 23.67	<0.001	13.49	12.15, 14.83	<0.001
Percent of the population identifying as Hispanic/Latino	19.08	15.24, 22.92	<0.001	15.87	13.93, 17.82	<0.001
Percent of the population that moved in since 2015	−4.54	−12.84, 3.77	0.280	−0.20	−4.6, 4.2	0.930
Percent of the housing that is vacant	7.35	−1.54, 16.25	0.110	6.57	1.91, 11.24	0.010
Percent of homes owned by the residents	−16.12	−20.17, −12.06	<0.001	−5.15	−7.02, −3.28	<0.001

## Data Availability

Publicly available datasets are available as follows: Alcohol outlet locations in NYC: NYS Liquor Authority Mapping Project (LAMP). https://sla.ny.gov. https://lamp.sla.ny.gov (accessed on 8 February 2021). Formerly redlined areas in NYC: Nelson RK, Winling L, Marciano R, Connolly N, et al., “Mapping Inequality,” American Panorama, 2017. ed. Robert K. Nelson and Edward L. Ayers. https://dsl.richmond.edu/panorama/redlining/ (accessed on 10 February 2021) [Bronx]. NYC violent crime data: Restrictions apply to the availability of the violent crime data. Although most of the data is available publicly (see: https://compstat.nypdonline.org (accessed on 8 February 2021), to protect the identity of victims, address-level locations of violent crimes are not publicly available. The data may be requested from the NYC Police Department. NYC demographics by US census block groups: Manson S, Schroeder J, Van Riper D, Kugler T, Ruggles S. IPUMS National Historical Geographic Information System: Version 16.0 [dataset]. Minneapolis, MN: IPUMS. 2021. http://doi.org/10.18128/D050.V17.0.
